# United States menhaden oil could save billions in U.S. health care costs and improve IQ in children

**DOI:** 10.1002/lite.201600008

**Published:** 2016-02-18

**Authors:** Douglas M. Bibus

**Affiliations:** ^1^ The University of Minnesota and Lipid Technologies LLCLipid Technologies, LLCPO BOX 216AustinMN55912

**Keywords:** fish oil, menhaden, supplements, Omega‐3, neural development, child IQ, cognitive health, cardiovascular disease, coronary heart disease

## Abstract

The United States menhaden oil annual production is sufficient to supply all of the recommended long chain Omega‐3s for Americans over 55 with coronary heart disease (CHD) and pregnant and lactating women. According to a recent study, the utilization of preventable intake levels could potentially save up to $1.7 billion annually in hospital costs alone. In addition, the remaining oil could be used to support a culture of enough Atlantic salmon to provide every pregnant and lactating woman in the U.S. with 8‐12 ounces of fish per week, as recommended by the Food and Drug Administration (FDA), throughout the duration of pregnancy and lactation. Based on the FDA's quantitative assessment, this may result in a net increase of IQ by 5.5 points in children and improve their early age verbal development.

The total United States medical expenditures are the highest in the world and have reached $ 2.8 trillion in 2012. Medical expenditures as a share of the Gross Domestic Product (GDP) have risen from 9.5% in 1985 to 17.2% in 2012, which is higher than in any other country. By 2039, it is expected to increase to 22%.

Cardiovascular disease (CVD) remains the leading cause of mortality in the U.S., causing more than 596,000 deaths annually. It costs the U.S. over $ 444 billion each year in total direct and indirect costs. Coronary heart disease (CHD) is a subset of CVD, killing over 375,000 people in the U.S. each year. Further, it costs the U.S. almost $ 109 billion each year in health care services, medications, and lost productivity. By 2030, the total costs are projected to increase to over one trillion U.S. dollars. However, this projection does not have to materialize, because fortunately, CVD is a partially preventable condition. The risk can be mitigated through healthy lifestyle choices and proper diet, including intake of adequate levels of Long‐Chain (LC) Omega‐3 fatty acids.

Published cost benefit analyses demonstrated that the utilization of LC Omega‐3 fatty acids at preventable intake levels among the target population may reduce the relative risk of a CHD event and result in potential net health savings 1, 2. Consistent with these reports, the American Heart Association (AHA) recommends that people with documented CHD consume 1,000 mg of eicosapentaenoic (EPA) and docosahexaenoic (DHA) acids per day, which is more than the typical Western diet provides.

A recent study conducted by the global consulting firm, Frost and Sullivan, estimated that nearly $ 1.7 billion in hospital costs could be avoided if all Americans over 55 with CHD consume the AHA‐recommended dose of LC Omega‐3s 3. Using the Frost and Sullivan's model, the U.S. annual menhaden oil production (74,700 MT/yr) could be used to supply all CHD patients in the U.S. with the recommended dosage of LC Omega‐3s (**Table** 1 and **Figure** 1), which could help realize these health cost savings.

**Table 1:** The United States produces enough menhaden oil to supply all Americans over 55 with CHD with recommended quantities of EPA and DHA. Additionally, the remaining oil could be used to help supply all pregnant and lactating women in the U.S. with recommended weekly servings of farm‐raised salmon. Furthermore, there would be enough oil left to supply 8 to 12 ounces of salmon per week to 5,962,677 to 13,336,650 individuals that would benefit from LC Omega‐3 consumption

Reference column	Metric	Measure	Note
A	U.S. menhaden oil production (10‐yr average), MT	74,700	11
B	EPA and DHA content of the oil, %	21,73%	12
C	Target population with CHD in the U.S.	17,016,536	3
D	Recommended intake of EPA and DHA for CHD patients, g/d	1	13
E	Oil needed to supply all U.S. CHD patients with the recommended dosage of EPA and DHA, MT	34,299	C*D*365/B/10^6*1.2 *
F	Oil left after supplying all CHD patients in the U.S., MT	40,401	A‐E
G	Inclusion rate of fish oil in salmon diets (2014), %	9%	5
H	Salmon feed conversion ratio	1.17	5
I	Salmon that could be produced from the leftover oil, MT	383,672	(((100 – G)/100*F/G) + F)/H
J	Salmon edible weight, %	68%	5
K	Edible salmon portions produced from the oil, MT	260,897	I*J
L	Recommended fish consumption (lower bound), oz/wk	8	6
M	Recommended fish consumption (upper bound), oz/wk	12	6
N	Weeks of salmon consumption at 8 oz/wk	1,150,339,784	K/0.00002835/L**
O	Weeks of salmon consumption at 12 oz/wk	766,893,189	K/0.00002835/M
P	Number of women that could be provided with three 8‐ounce portions of salmon each week for a year	22,121,919	N/52
Q	Number of women that could be provided with two 12‐ounce portions of salmon each week for a year	14,747,946	O/52
R	Number of pregnancies in the U.S.	6,369,000	7
S	Number of live births in the U.S.	4,131,000	7
T	Average duration of pregnancy, wks	38	14
U	Recommended duration of lactation, wks	52	15
V	Total number of pregnant and lactating women in the U.S. to be supplied with salmon for a year	8,785,269	R*T/52 + S

* The 1.2 coefficient was used to adjust for the 17% yield loss on oil refining before it becomes palatable for direct human consumption.

** The 0.00002835 coefficient for the conversion of metric tons to ounces.

**Figure 1:**
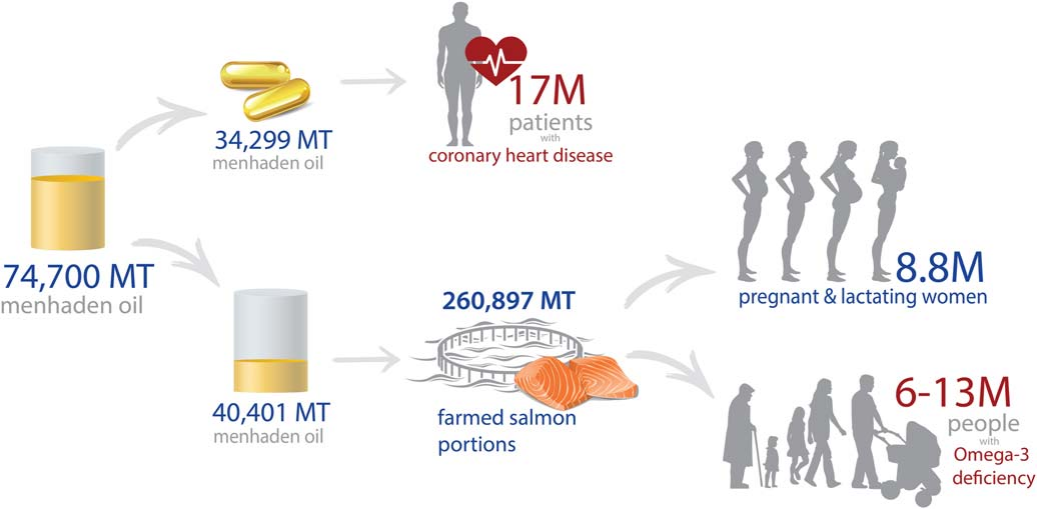
U.S. menhaden production is sufficient to supply omega‐3s for americans over 55 with coronary heart disease, pregnant and lactating women, and other deficient populations.

These potential net health savings are probably only the tip of the iceberg of what could be attained if all of U.S. menhaden oil was used to provide the AHA‐recommended amount of LC Omega‐3s to Americans over 55 with CHD. The model does not account for other CHD‐related expenditures, like cost of lost productivity, medications, and most importantly, value of lives saved. Additionally, it does not capture the savings derived from positive health effects of LC Omega‐3s on other subsets of CVD, which frequently accompany CHD in adults over age 55. For example, it is well‐documented that LC Omega‐3s may have blood pressure benefits. The costs associated with hypertension in the U.S. exceed $ 93.5 billion a year 4. Finally, the model does not account for the healthcare savings that could result from the impact of fish oil on non‐CVD‐related conditions, like cognitive impairment, which affects approximately 16 million people in the U.S.

Besides cardiovascular health, LC Omega‐3s support neural development and function. DHA is a predominant structural fatty acid in the central nervous system and retina. It cannot be efficiently synthesized by humans from shorter‐chain Omega‐3s and is best obtained through the diet, especially during rapid brain growth and fetal neural development.

Recently, the Food and Drug Administration (FDA) published a quantitative assessment of the impact of fish consumption on fetal neurodevelopment 15. The agency reported that adequate fish consumption by mothers during pregnancy can result in a net increase of IQ by 5.5 points in children and improve their early age verbal development 15. It is recommended that women who are pregnant, might become pregnant, or are breastfeeding, consume 8 to 12 ounces of fish per week 15.

In addition to the U.S. yearly menhaden oil production that could be supplied directly to people with CHD, on average there would be 40,401 MT of oil left for other uses. As salmon feeds currently contain up to 9% fish oil 5, and the current feed conversion ratio is 1.17 5, the leftover oil could be used to support the production of 383,672 MT of farmed salmon. After the adjustment for 68% edible yield 5, this equates to 260,897 MT of edible salmon portions. This quantity is sufficient to supply every pregnant and lactating woman in the U.S. with two to three servings of oily fish per week, as recommended by the FDA 6. The remaining salmon can be supplied to 5,962,677 to 13,336,650 additional consumers (e.g. 14,747,946 – 8,785,269 = 5,962,677; 22,121,919 – 8,785,269 = 13,336,650) that would benefit from the increased LC Omega‐3 consumption.

This is a very conservative estimate for two reasons. First, we assume that all women who become pregnant in a given year are consuming the recommended amount of fish regardless of the outcome of the pregnancy (only 65% of the pregnancies result in live birth) 7. Second, we assume that all infants are breastfed for a whole year, as recommended by the American Academy of Pediatrics. According to FDA, the adequate intake of LC Omega‐3 fatty acids may result in a net increase of IQ of up to 5.5 points in children by the time they reach the age of eight 6. Theoretically, over time, this could increase the average IQ of the U.S., moving it higher in international rankings from its current place at 19th 8, 9.

Although the remaining 32% of farm‐raised salmon weight is considered non‐edible in most of the Western world, the byproducts are typically processed into the feed ingredients for other fish and shrimp species, food animals, laboratory and zoo animals, pets, and humans through salmon oil supplements. As to aquaculture and salmon farming in particular, providing the LC Omega‐3s to humans by including the oil from small pelagic species in farmed fish diets is a far more efficient method of harnessing the ocean's healthy nutrients than harvesting larger food fish 10. This is because, in nature, one unit of salmon biomass increase would require at least ten units of small pelagic prey fish 10. However, as demonstrated by Bibus, only less than 0.8 units of menhaden was required in 2013 to grow one unit of farmed salmon because marine ingredients today represent only a small fraction of the feed by weight 11. Further, as the inclusion rates of oil in salmon feed have declined, a simple calculation using Bibus' approach shows that in 2014 it would take only 0.7 units of menhaden to produce one unit of salmon. Thus, the leftover LC Omega‐3s continue to serve its role in human and animal nutrition, as market forces do not allow these nutrients to be wasted.

## Conclusions

Although not fully quantifiable at this time, the potential impact of our fish oil production on the U.S. medical expenditures is astounding. The magnitude of the actual current impact on healthcare costs across various health conditions may already be close to that of the theoretical, as almost 75% of U.S.‐produced menhaden oil is currently used in production of farmed salmon and supplements, which are the means of transferring marine LC Omega‐3s through the food chain to humans. However, the U.S. menhaden oil production should not be evaluated merely on its ability to reduce healthcare costs. Instead, it should be valued based on a combination of cost savings and contribution to human well‐being, including healthy life expectancy and quality of life.
